# An uncommon and challenging finding regarding the tricuspid valve: case report, clinical considerations, and practical management

**DOI:** 10.1093/ehjcr/ytae474

**Published:** 2024-09-06

**Authors:** Edoardo Sciatti, Raul Limonta, Salvatore D’Isa, Vincenzo Duino, Michele Senni

**Affiliations:** Cardiology Unit, ASST Papa Giovanni XXIII, Piazza OMS 1, 24127 Bergamo, Italy; School of Medicine and Surgery, University of Milan-Bicocca, Piazza dell'Ateneo Nuovo 1, 20126 Milan, Italy; Cardiology Unit, ASST Papa Giovanni XXIII, Piazza OMS 1, 24127 Bergamo, Italy; Cardiology Unit, ASST Papa Giovanni XXIII, Piazza OMS 1, 24127 Bergamo, Italy; Cardiology Unit, ASST Papa Giovanni XXIII, Piazza OMS 1, 24127 Bergamo, Italy; School of Medicine and Surgery, University of Milan-Bicocca, Piazza dell'Ateneo Nuovo 1, 20126 Milan, Italy

**Keywords:** Tricuspid, Mass, Aneurysm, Case report

## Abstract

**Background:**

The differential diagnosis of tricuspid masses remains challenging.

**Case summary:**

This case involves the incidental detection of a lesion with a non-solid appearance, exhibiting the characteristic ‘finger-in-glove’ and ‘garland-like’ morphology, resembling a blind-ended protrusion of the tricuspid leaflet. This presentation is consistent with a tricuspid valve aneurysm, without significant associated stenosis or regurgitation.

**Discussion:**

Given the lesion’s morphological features, the patient’s asymptomatic status, and the absence of a precipitating event suggestive of an alternative diagnosis, we concluded that the most likely diagnosis is aseptic tricuspid valve aneurysm. Following a multidisciplinary heart team discussion, surgical intervention was deemed unnecessary.

**Learning points**

- The differential diagnosis of tricuspid valve masses is still challenging.
- Valve aneurysm are non-solid lesions, with the typical ‘finger-in-glove’ and ‘garland-like’ appearance, similar to a blind-ended protrusion of the leaflet; they are generally associated to endocarditis.

## Introduction

The differential diagnosis of tricuspid masses is still challenging, due to technical imaging difficulties and the wide spectrum of lesions. Valvular aneurysms are rare conditions, generally associated with endocarditis, and their treatment depends on their impact on valvular function.^1^ In individuals undergoing transoesophageal echocardiogram (TEE), mitral valve aneurysms (MVA) have been previously documented at a rate of 0.2–0.29%, with a notably higher prevalence of involvement of the anterior leaflet compared to the posterior one, often associated with infective endocarditis.^2^ In the absence of any other cardiac or systemic pathology, degenerative changes related to the aging process are believed to underlie leaflet failure and the development of MVA, as elevated left ventricular (LV) pressure leads to the protrusion of the mitral valve leaflet into the left atrium. Conversely, tricuspid valve aneurysms (TVAs) have been less frequently reported in the medical literature, generally associated with the rupture of a valve abscess complicating a previous episode of infective endocarditis.^3,4^

## Summary figure

**Figure:**
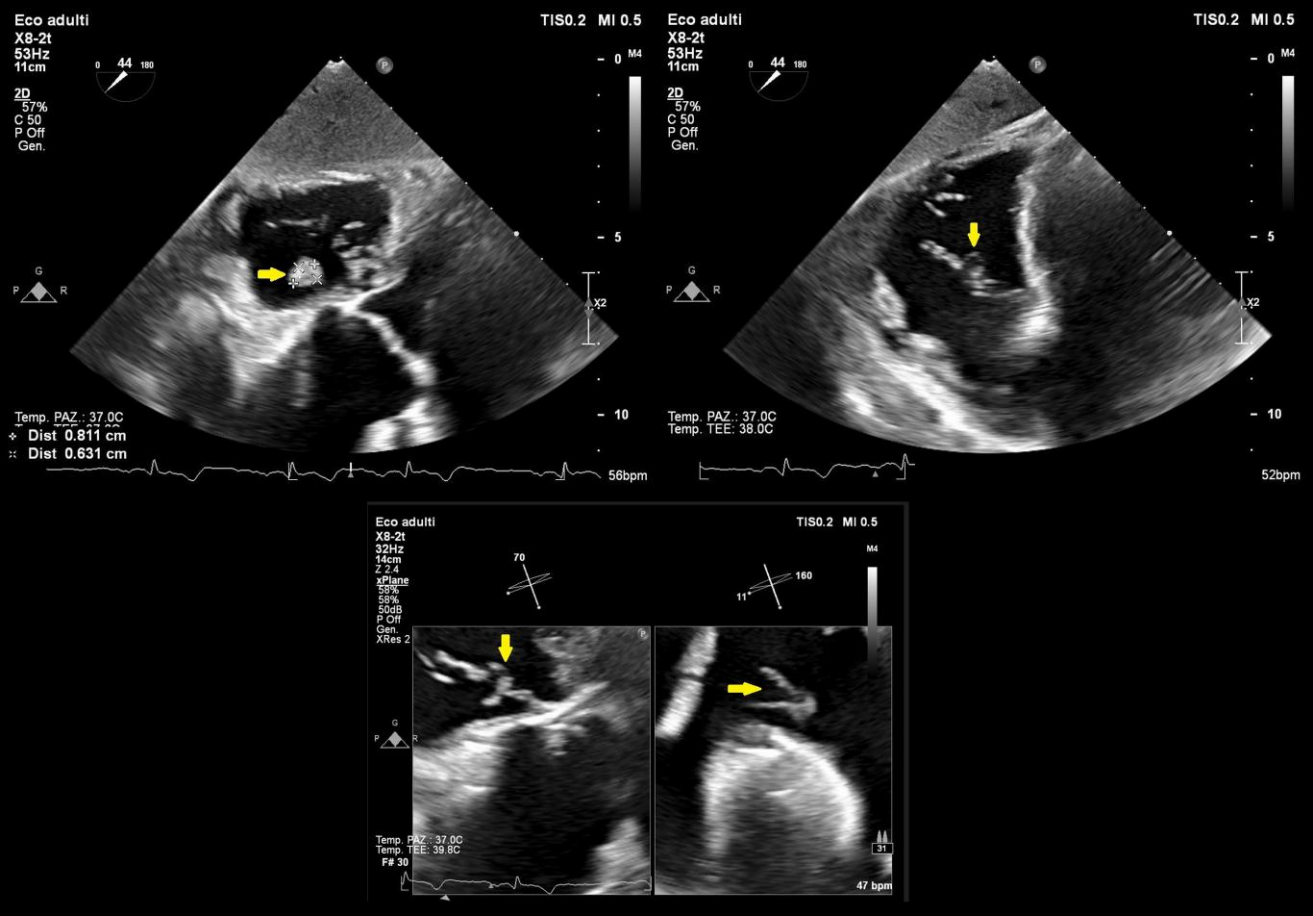
Different short axis views of the tricuspid valve from the transoesophageal transgastric window, showing the tricuspid valve aneurysm.

## Case presentation

**Figure 1:**
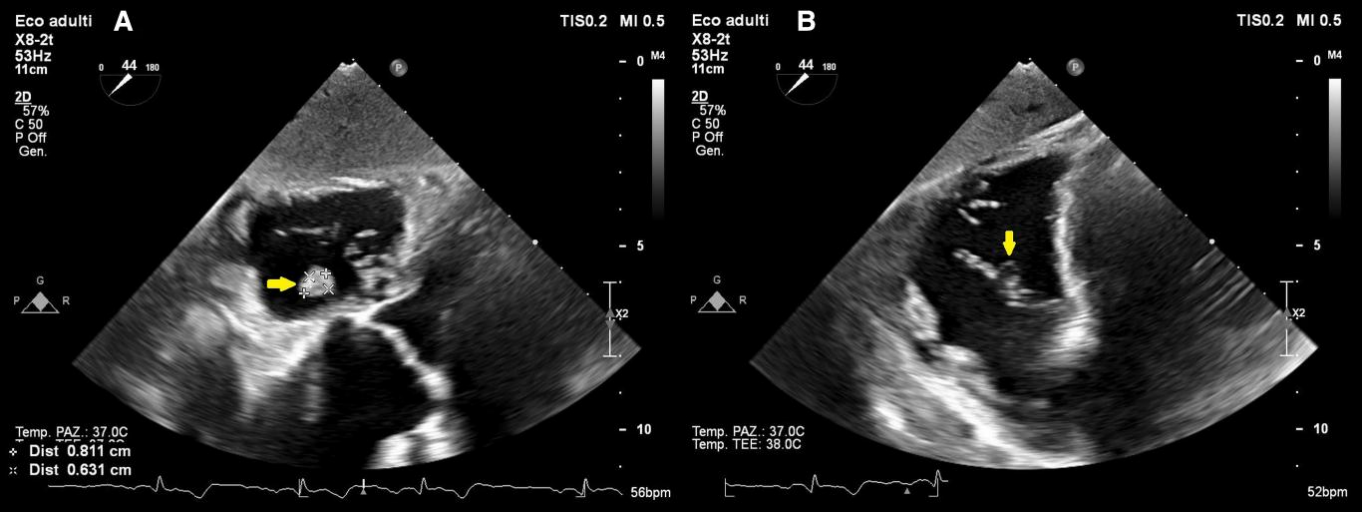
(*A*) Short axis view of the tricuspid valve from the transoesophageal transgastric window, showing an oval hyperechoic mass attached to the anterior leaflet. (*B*) Off-axis view of the same window revealing an internally empty mass.

**Figure 2:**
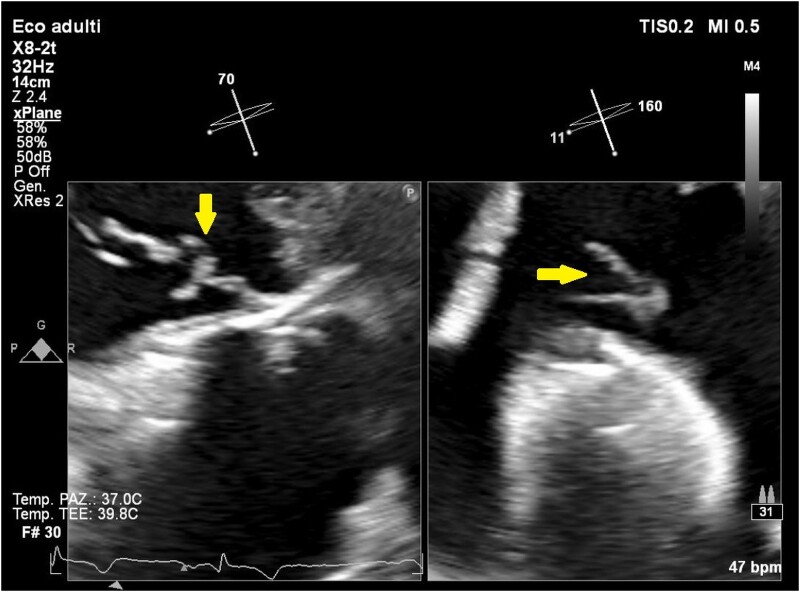
Zoomed off-axis short axis view of the tricuspid valve from the transoesophageal transgastric window with X-plane imaging, showing a ‘finger-in-glove’ and ‘garland-like’ appearance with adherent filaments resembling second-order chordae tendineae.

**Figure 3:**
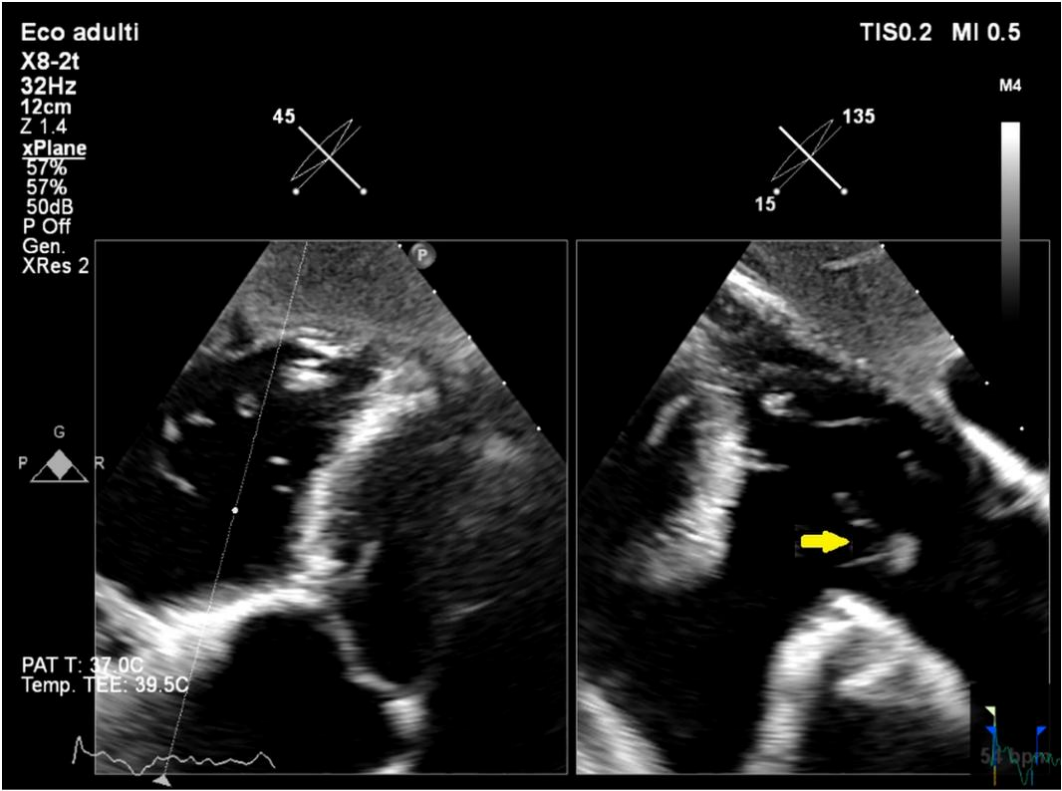
Off-axis short axis view of the tricuspid valve from the transoesophageal transgastric window with X-plane imaging, showing a ‘finger-in-glove’ and ‘garland-like’ appearance with adherent filaments resembling second-order chordae tendineae.

**Figure 4:**
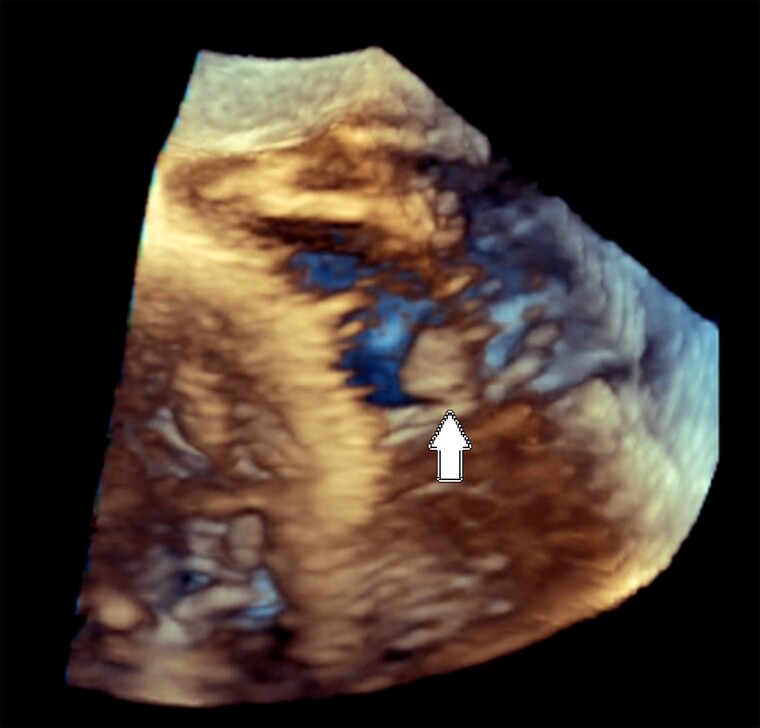
3D elaboration of the tricuspid valve from the transoesophageal transgastric window showing an oval mass attached to the anterior leaflet. The resolution is not enough to catch the true nature of the lesion.

We present the case of a 74-year-old man who was referred to our outpatient Heart Failure Clinic after discharge for antero-septal ST-segment elevation myocardial infarction with LV systolic dysfunction, managed through primary percutaneous coronary intervention and two drug-eluting stents implantation from the ostial left main coronary artery to the left anterior descending artery. The patient’s medical history was consistent for dyslipidaemia, overweight (BMI 25.8 kg/m²), active smoking habit, and a previously surgically treated infra-renal abdominal aortic aneurysm. Cardiovascular and respiratory exam did not find any clinical signs. Resting transthoracic echocardiogram (TTE) revealed mild LV dilation (EDD 57 mm) with eccentric hypertrophy (IVST 10 mm, PWT 8 mm) and moderate pump dysfunction (LVEF 46%) due to akinesia of the anterior and mid-septal walls and all apical segments, second-degree diastolic dysfunction (E/A 1.4, E/e′ 12). Left atrium was severely dilated (LAVi 48 mL/m²). Both aortic cusps and mitral leaflets appeared sclerotic with concomitant mild aortic and moderate mitral regurgitation. The right ventricle was not dilated and normokinetic (TAPSE 24 mm, S′ 11 cm/s). Also right atrium was not dilated. Tricuspid regurgitation was mild with a derived pulmonary artery systolic pressure of 52 mmHg. Inferior vena cava was normal and collapsible and pericardial effusion absent. Additionally, the aorta displayed mild dilation at the level of the sinuses of Valsalva, along with an aneurysmatic ascending tubular tract (46 mm), and the aortic arch exhibited diffuse atherosclerotic changes. Notably, the resting TTE revealed the presence of an ∼9 mm round, hyperechoic mass adherent to the ventricular surface of the anterior tricuspid valve leaflet, showing systolic excursion towards the right atrium (see Supplementary material online, *Video S1*). Therefore, the patient underwent TEE for a more comprehensive assessment of the tricuspid mass. Transoesophageal echocardiogram confirmed a structure measuring ∼6 × 8 mm attached to the ventricular surface of the tricuspid anterior leaflet (*Figure 1*, Supplementary material online, *Video S2*–*S4*). Specifically, this lesion presented a non-solid aspect, bearing the typical ‘finger-in-glove’ appearance, similar to a blind-ended protrusion of the tricuspid leaflet itself. The structure seemed internally empty, with adherent filaments resembling second-order chordae tendineae, exhibiting a ‘garland-like’ appearance. In terms of echogenicity, its appearance closely resembled that of the adjacent valvular tissue, suggesting that it was, in fact, in close continuity. Moreover, it moved in relation to the cardiac cycle, ‘bulging’ like a sail during systole and relaxing during diastole (*Figures 2–4*, Supplementary material online, *Video S5*–*S8*). While the patient remained entirely asymptomatic, we opted to perform a contrast-enhanced chest CT scan to exclude pulmonary embolism, given the propensity of many cardiac masses to embolize distally, which yielded a negative result. Blood cultures were also negative. At 6 months, the patient’s clinical status was unremarkable, with TTE showing findings similar to those observed previously, and a contrast-enhanced chest CT scan ruled out new distal embolizations. In order to further characterize the lesion, the patient was offered cardiac magnetic resonance, which he declined due to claustrophobia. A follow-up programme is still ongoing.

## Discussion

Differential diagnosis included many cardiac masses.^5^ The lesion location and aspect exclude the potential diagnosis of a myxoma or, even less likely, a cardiac sarcoma. Myxomas typically originate in the atria, with a preference for the left atrium, present as well-defined, mobile masses with stalk-like attachments and variable gelatinous or myxoid shapes and exhibit a characteristic swinging motion known as the ‘tumour plop’ coinciding with cardiac cycles and may cause symptoms like syncope, dyspnoea, or chest pain.^5^ Cardiac sarcomas often present as dense, irregular, nonmobile masses located in the left atrium, extending from the endocardium to the myocardium. They are also associated with advanced symptomatic metastatic disease.^5^ Fibroelastoma, generally attached to valve leaflets, however typically presents with a more irregular and less clearly demarcated appearance than what we have observed.^5^ Furthermore, considering the absence of symptoms and distal embolization, we maintain a high degree of confidence in excluding the possibility of it being a cardiac tumour. In addition, cardiac thrombi or vegetations were considered. Our patient’s tricuspid lesion was completely different in aspect. Endocardial thrombus typically appears as echodense structures contrasting with the surrounding blood, while endocarditis vegetation predominantly affects the mitral and aortic valves and manifests as irregular, echogenic masses firmly adhering to valve leaflets, chordae tendineae, endocardial surfaces, or even on device leads. Our patient’s tricuspid lesion appeared as an empty structure with internal filamentous elements resembling taut chordae, in contrast to the typical characteristics of thrombi and vegetations. Furthermore, the patient did not meet any of the Duke criteria, leading to the consideration that the diagnosis of infective endocarditis was unlikely. Additionally, considering the absence of any risk factors that could predispose to the formation of an intracavitary thrombus or endocarditis, we dismiss the idea of it being a thrombus or a consequence of a previous infection of the leaflet itself. The lesion strongly resembled the extremely rare TVA, which generally are associated to a previous episode of endocarditis.^1,3^ Only another case in literature was not associated to infection.^4^ We are confident to think that our case of TVA was not a consequence of a recent infective endocarditis, as no relevant medical history supported such an association, and blood cultures yielded negative results. Libman–Sacks endocarditis was also ruled out due to the absence of a history of cancer or autoimmune disease. However, it is important to emphasize that the possibility of a previous subclinical endocarditis with a favourable course, i.e. without significant tricuspid regurgitation, cannot be definitively ruled out in the absence of current signs of infection or other suggestive imaging features.

In light of its characteristics, the patient’s asymptomatic state, and the absence of a precipitating event that would suggest a specific diagnosis, we concluded in favour of a saccular dilation of the septal tricuspid leaflet compatible with TVA, most likely of aseptic nature, for which, following a heart team discussion, it was not considered beneficial to surgically intervene. Furthermore, given our prior experience with similar lesions of the mitral valve,^6^ we felt confident enough to avoid further diagnostic investigations, such as cardiac magnetic resonance imaging, which is instead the current gold standard for the differential diagnosis of cardiac masses.

## Lead author biography


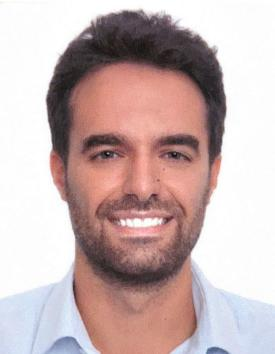


Dr Edoardo Sciatti works at the Heart Failure Clinic of ASST Papa Giovanni XXIII in Bergamo, Italy, and is involved in several international trials about HFrEF and HFpEF therapy. His main interests beyond heart failure are valvular heart diseases, echocardiography in interventional cardiology, and cardiovascular complications of pregnancy.

## Supplementary Material

ytae474_Supplementary_Data

## Data Availability

The data underlying this article will be shared on reasonable request to the corresponding author.

## Notes

**Consent:** The authors confirm that written consent for submission and publication of this case report including the images and associated text has been obtained from the patient in line with COPE guidance.

**Funding:** None.
